# Supplementary material for: Hysteresis stabilizes dynamic control of self-assembled army ant constructions
Source: Nat Commun. 2022 Mar 4;13:1160. doi: 10.1038/s41467-022-28773-z (PMC8897433; doi:10.1038/s41467-022-28773-z)
Supplement: Supplementary file 1 — Supplementary Information [file 41467_2022_28773_MOESM1_ESM.pdf]

Supplementary Information: Hysteresis stabilizes dynamic control of self-assembled army ant constructions

Helen F. McCreery[a,1], Georgina Gemayel[b], Ana Isabel Pais[c,d], Simon Garnier[c], and Radhika Nagpal[a,e]

[a] School of Engineering and Applied Sciences, Harvard University
[b] University of Southern California
[c] Department of Biological Sciences, New Jersey Institute of Technology
[d] Rutgers University, Newark
[e] Wyss Institute for Biologically Inspired Engineering

[1] Corresponding author; e-mail: hmccreery@gmail.com

Supplementary Note 1: Details of structural data extraction

Extracting side- and top-view envelopes: We automatically extracted the bridge envelopes from the side and top videos of experiments for each second of each trial using Matlab. In order to exclude ants crossing the bridge from the envelope and include only the bridge itself, we first averaged video frames over 10 seconds (including 5 seconds before and 5 seconds after the focal frame), as shown in Figure S1, panel 1. Ants walking on a bridge then appear as a faint blur, and are excluded from the automatically generated envelope. The typically high contrast between the ant bridges and both the platforms and the green cardstock (which we affixed in the background) allowed us to use simple hue-saturation-value (HSV) thresholding to distinguish the bridge structure from the surroundings, with each pixel assigned either as part of an ant, or not (Supplementary Figure 1, panels 2 and 3). We manually chose separate HSV thresholds for each of the 10 complete trials, as the lighting conditions differed.

This process produced relatively noisy bridge envelopes (Supplementary Figure 1, panel 4), which often included multiple segments which were actually part of a continuous bridge (because the connecting piece, such as a single leg between the two bridge sections was not picked up). We combined bridge segments that were sufficiently close together using the concaveman package[1] in R, which produces “concave hulls” (Supplementary Figure 1, panel 5). We had some additional smoothing steps (using the smoother package[2]) to produce more accurate envelopes, including gaussian smoothing of the final shapes (Supplementary Figure 1, panel 6). The smoothing and remaining analyses were all conducted in R.

Estimating metrics for 3D structures: After extracting the side- and top-envelopes of bridges over time, we combined these data to extract metrics about the 3D structures, as described in Figure 1 of the main text. We used the sf package[3] in R, for spatial data, as well as the tidyverse package[4]. As the distance between our cameras and the apparatus was not uniform across trials, we first scaled the envelopes to be in known, global units (where 100 scaled “pixel” units = 1 cm), using the locations of fixed points on the platforms. Likewise, we used these fixed points to place the side and top views into the same coordinate system. Because the side and top videos were not started at exactly the same time, we also shifted the time such that we always focused on side and top envelopes from the same

focal second (having separately used the audio from the side and top recordings to automatically calculate the correct delay using Matlab).

For several metrics, we needed to step through “slices” of the bridge structures, in the dimension running along the length of the bridge (x-dimension). Slices were one pixel-unit wide (one tenth of one mm). As shown in Figure 1 in the main text, for each x position, we recorded the width of the bridge (width of top envelope, w_x), the height (height of side envelope, h_x), and the vertical midpoint of the slice (midpoint of vertical line through side envelope). We define the midline of the bridge by connecting these midpoints. We approximated the shape of these slices as ellipses, therefore for a particular x position, the area of the slice is equal to $\pi w_x h_x$. As each slice is one unit thick, the volume is equal to the area, and we therefore calculate the **volume** of the bridge by summing the volumes of all of the slices. The **mean cross-sectional area** is simply the mean of the slice areas (also equal to the volume divided by the length of the midline). We measured the tautness of bridges by measuring the relative **height of the center of mass**, using the height of the centroid (center of gravity) of the side envelope. Lower values indicate a more slack bridge.

Supplementary Note 2: Optical flow extraction and analyses

Extracting optic flow: We used relative optical flow on the bridge vs platforms as a measure of traffic performance. To do so, we first automatically extracted dense optical flow from top-view videos of experiments (main text Figure 5) using Farnebäck's algorithm[5] as implemented in OpenCV[6] (version 4.3) using all default parameters and a window size of 5 pixels. Optical flow measures the apparent motion of objects between two consecutive frames in a sequence of images. Thus, it can be used to estimate the flow rate of ants over the experimental platform and the bridge.

Before measuring the optical flow in our videos, we rescaled and lined up all the video frames so that (1) the width of the adjustable platform was always 200 pixels (100 pixels = 1 cm), and (2) the location at which the fixed and adjustable platforms meet was always the same and could therefore be used as a standardized origin of the coordinate system across all videos. We then calculated the optical flow between all consecutive frames of each video, separating left- and right-bound traffic using the direction of the optical flow vectors relative to the orientation of the experimental apparatus. Moreover, left- and right-bound optical flow vectors across the width of the platforms were averaged along the main axis of the experimental apparatus (one average value for each column of pixels). Finally, optical flow over the bridge and the platforms were averaged separately over consecutive 10-seconds periods.

The contrast between moving ants on the platforms (which are white) and the bridge (which is darker) does not allow for direct comparison between optical flow measured on both surfaces. Instead, we performed separate least-squares regressions of bridge flow on platform flow for each trial. The signed residuals from these regressions can, therefore, be interpreted as a measure of the traffic performance of the bridge, relative to the incoming traffic from the platform. Computing performance separately for each trial allowed us to account for trial-level differences in optical flow measurements, for example due to different lighting conditions.

Sensitivity analysis: As described in “Methods: Data analysis” in the main text, we measured performance every 10 seconds. We tested and confirmed that our conclusions are not sensitive to this choice by varying this frequency. Performance in the 5 seconds, 20 seconds, and 30 seconds before

joining events are all significantly higher than expected, though for larger intervals the effect size decreases (see SI Figure S14).

Because events rarely occurred precisely between two 10-second intervals for which we had performance data, assigning *which* interval precedes each event was not trivial. If an event occured within the latter half (latter 5 seconds) of a performance interval, we used that interval as the preceding event; if the event occurred within the first half, we used the *previous* interval. The analysis is not sensitive to this choice; our conclusions are the same if we are more conservative about what counts as the preceding interval (for example, counting an interval as preceding an event only if the event occurs in the last 2 seconds or 1 second of an interval, or even if we only look at the last interval before events which never contain the event).

Supplementary Figures

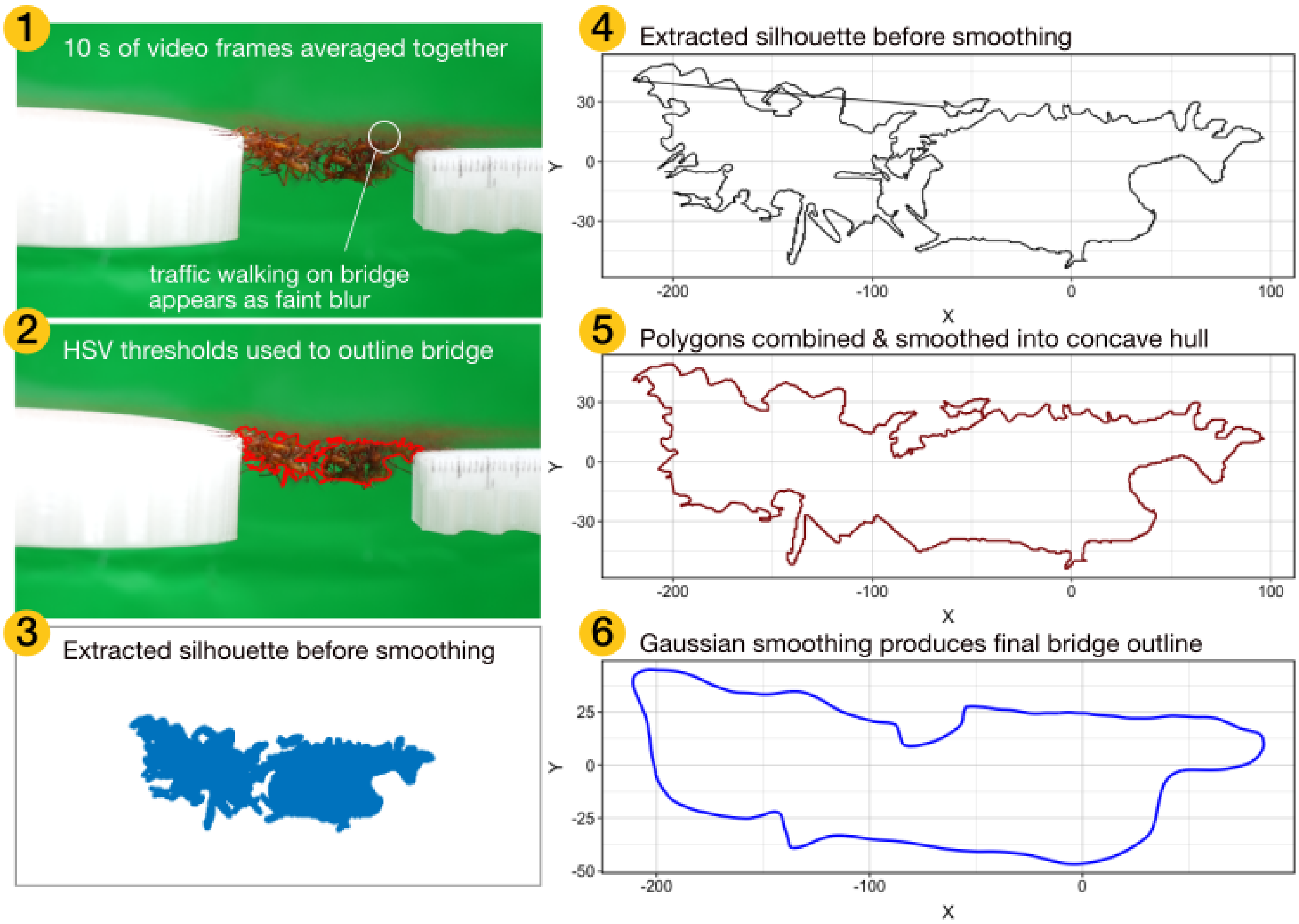

Supplementary Figure 1. Steps to extract side- and top-view envelopes of bridges. Steps shown in panels 1 and 2 completed in Matlab, panel 3 shows the output from Matlab. Steps shown in panels 4 through 6 performed in R. Details described Appendix A2: Extracting side- and top-view envelopes.

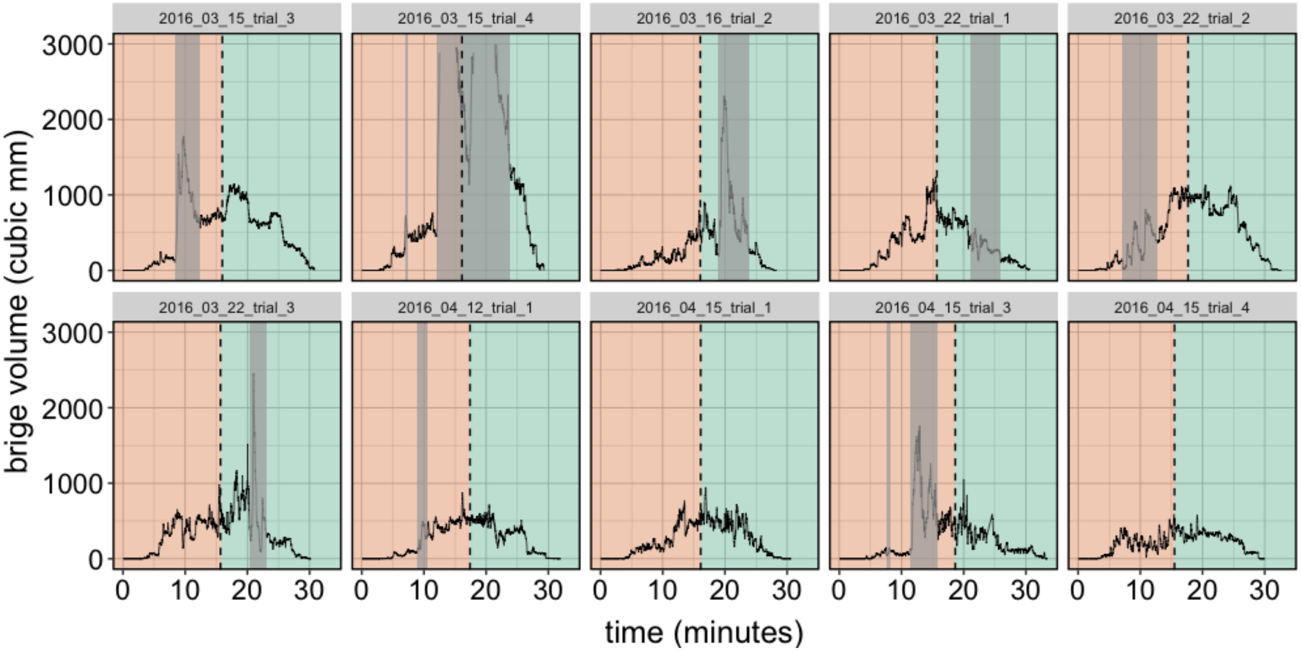

Supplementary Figure 2. Volume of self-assembled bridges for all trials. Dotted vertical line indicates the time when the experiment shifted from the expansion phase (increasing gap size) to contraction phase (decreasing gap size). Gray shading indicates that the bridge was broken or recovering from a break; results metrics may be inaccurate during these periods.

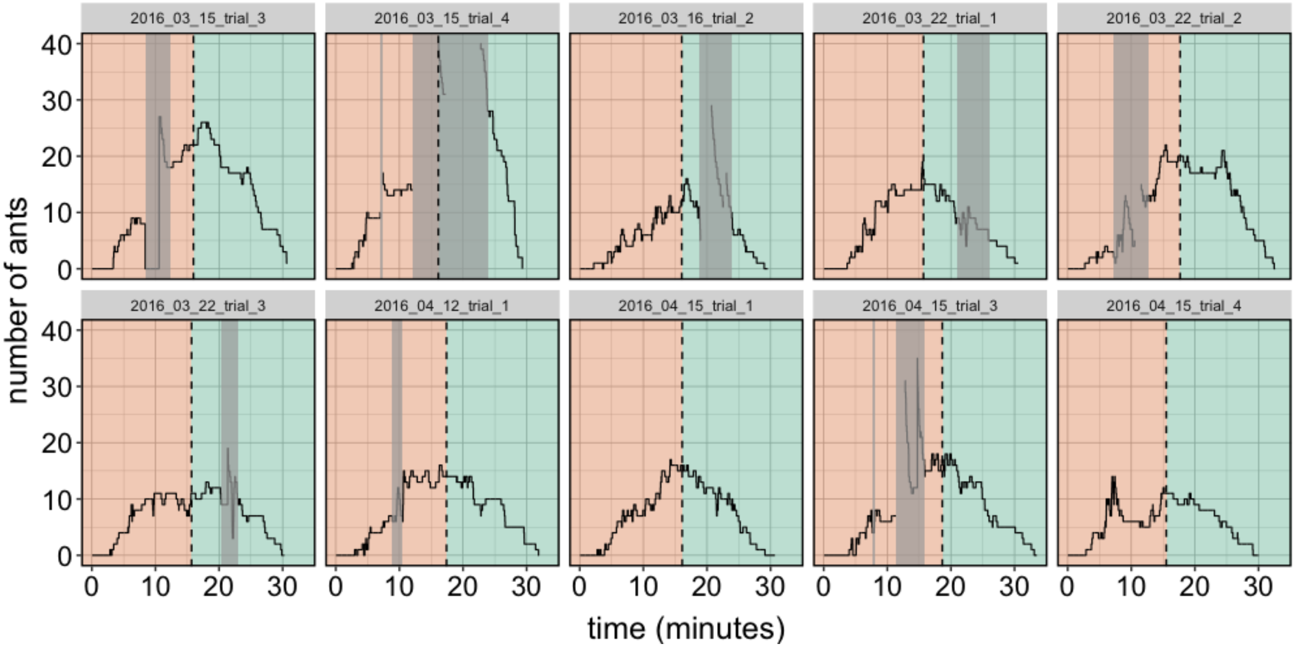

Supplementary Figure 3. Number of ants in self-assembled bridges for all trials. Dotted vertical line indicates the time when the experiment shifted from the expansion phase (increasing gap size) to contraction phase (decreasing gap size). Gray shading indicates that the bridge was broken or recovering from a break; results metrics may be inaccurate during these periods.

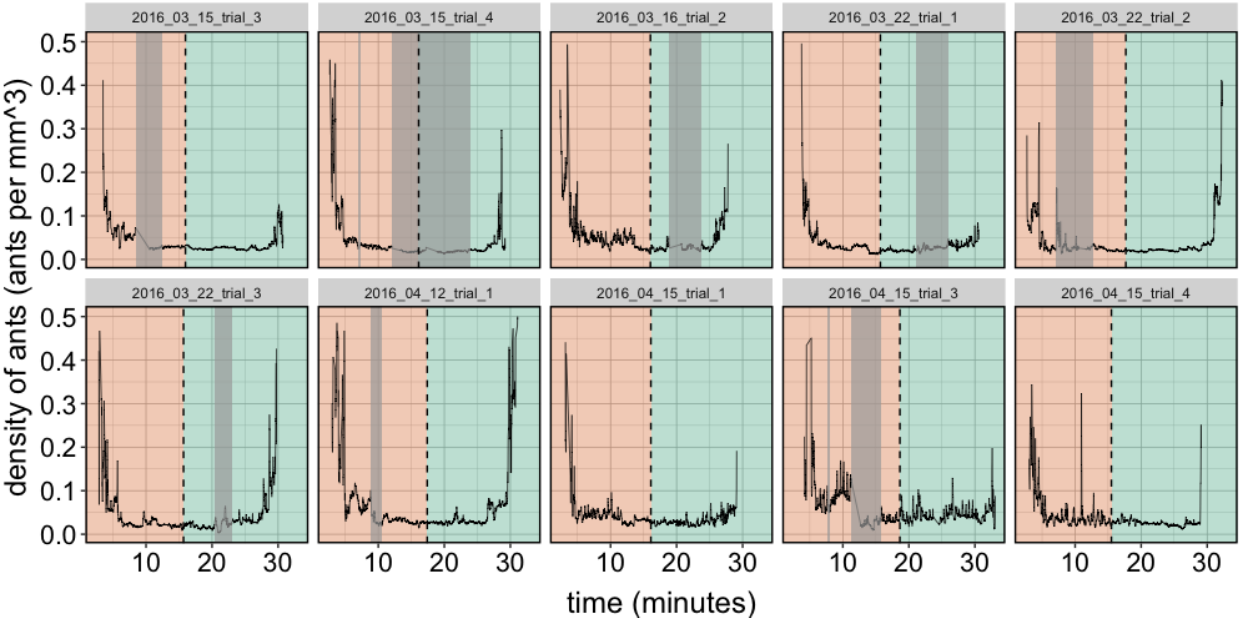

Supplementary Figure 4. Density (ants per cubic mm) is consistent through time and across trials. The Pearson correlation coefficients for the volume and number in the bridge for each trial are 0.97, 0.96, 0.91, 0.91, 0.98, 0.88, 0.95, 0.93, 0.92, and 0.89, respectively. There tend to be extreme values of density at the beginnings and ends of trials, when bridges are fleeting, such density values above 0.5 are excluded for clarity. While volume, and thus density, is relatively noisy, values are relatively consistent in stable bridges. Dotted vertical line indicates the time when the experiment shifted from the expansion phase (increasing gap size, in orange) to contraction phase (decreasing gap size, in green). Gray shading indicates that the bridge was broken or recovering from a break; results metrics may be inaccurate during these periods.

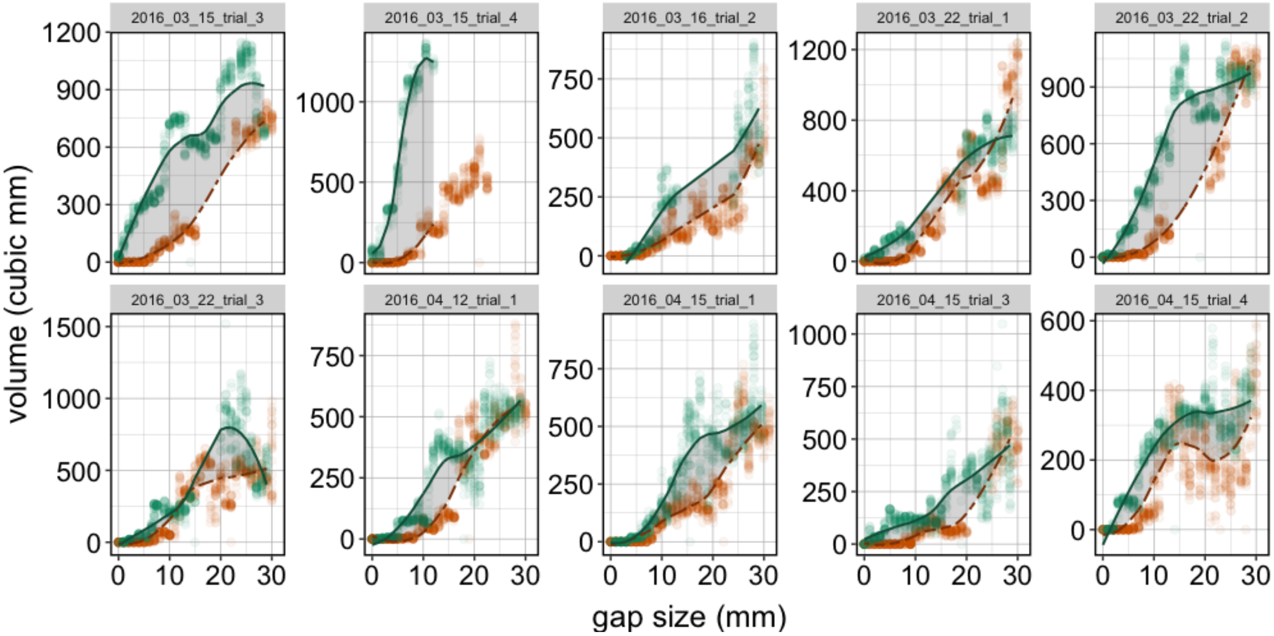

Supplementary Figure 5. Hysteresis in volume for all trials. Trials show hysteresis, with volume at a particular gap size differing during the expansion and contraction phases. Points show individual measurements, taken every second; periods when the bridge was broken or recovering are excluded. Lines are smoothed LOESS (local regression) for the expansion (orange points, dashed orange line) and contraction (green points, solid green line) phases. The area between the smoothed lines (shaded gray) shows the extent of hysteresis. Points are jittered to improve clarity.

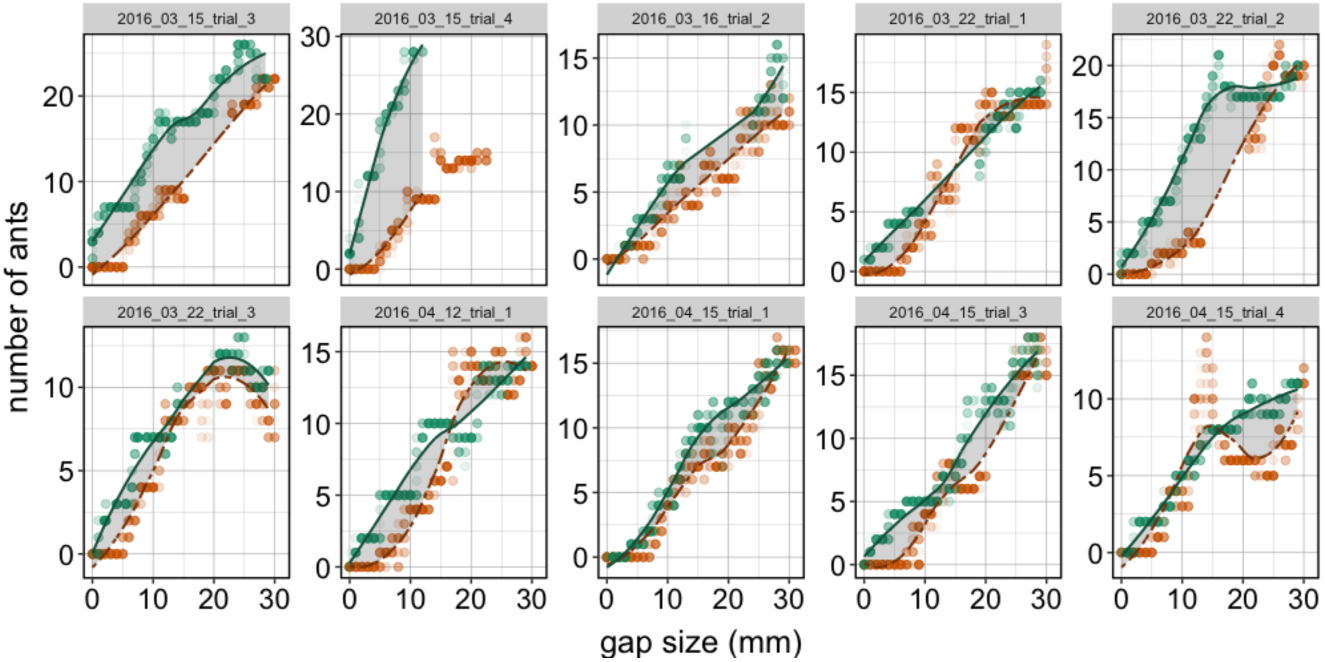

Supplementary Figure 6. Hysteresis in number of ants for all trials. Trials consistently show hysteresis, with the number of ants making up a bridge at a particular gap size differing during the expansion and contraction phases. Points show individual measurements, taken every second; periods when the bridge was broken or recovering are excluded. Lines are smoothed LOESS (local regression) for the expansion (orange points, dashed orange line) and contraction (green points, solid green line) phases. The area between the smoothed lines (shaded gray) shows the extent of hysteresis.

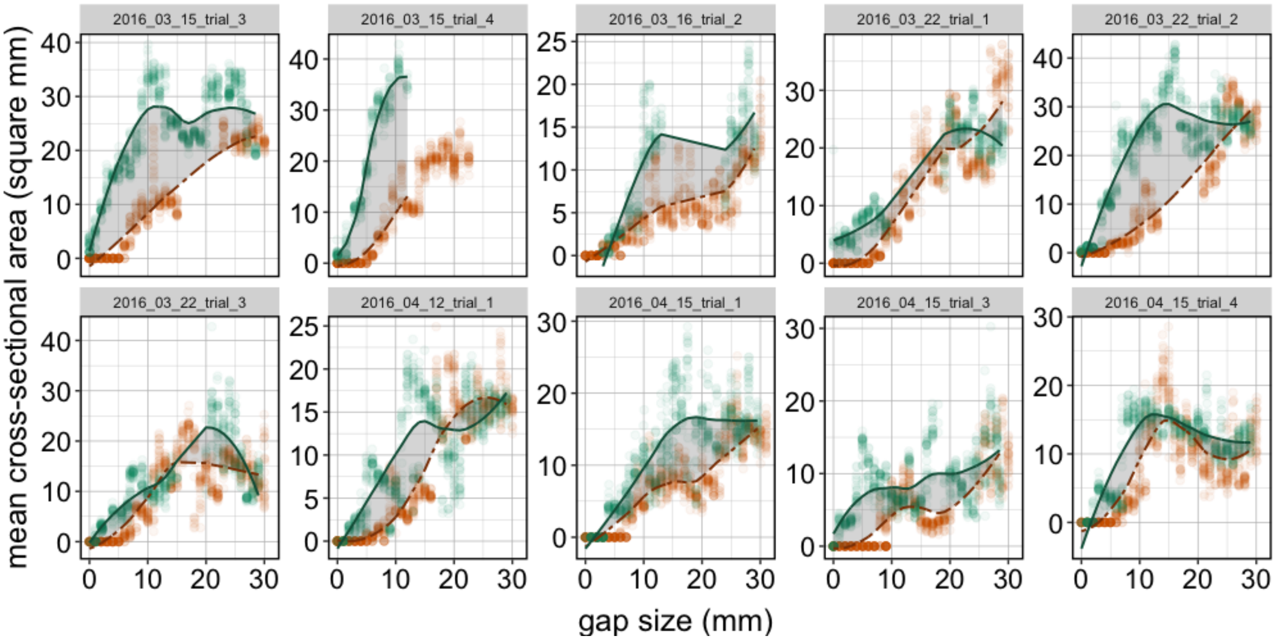

Supplementary Figure 7. Hysteresis in mean cross-sectional area for all trials. Trials consistently show hysteresis, with the cross-sectional area of a bridge at a particular gap size differing during the expansion and contraction phases. Points show individual measurements, taken every second; periods when the bridge was broken or recovering are excluded. Lines are smoothed LOESS (local regression) for the expansion (orange points, dashed orange line) and contraction (green points, solid green line) phases. The area between the smoothed lines (shaded gray) shows the extent of hysteresis. Points are jittered to improve clarity.

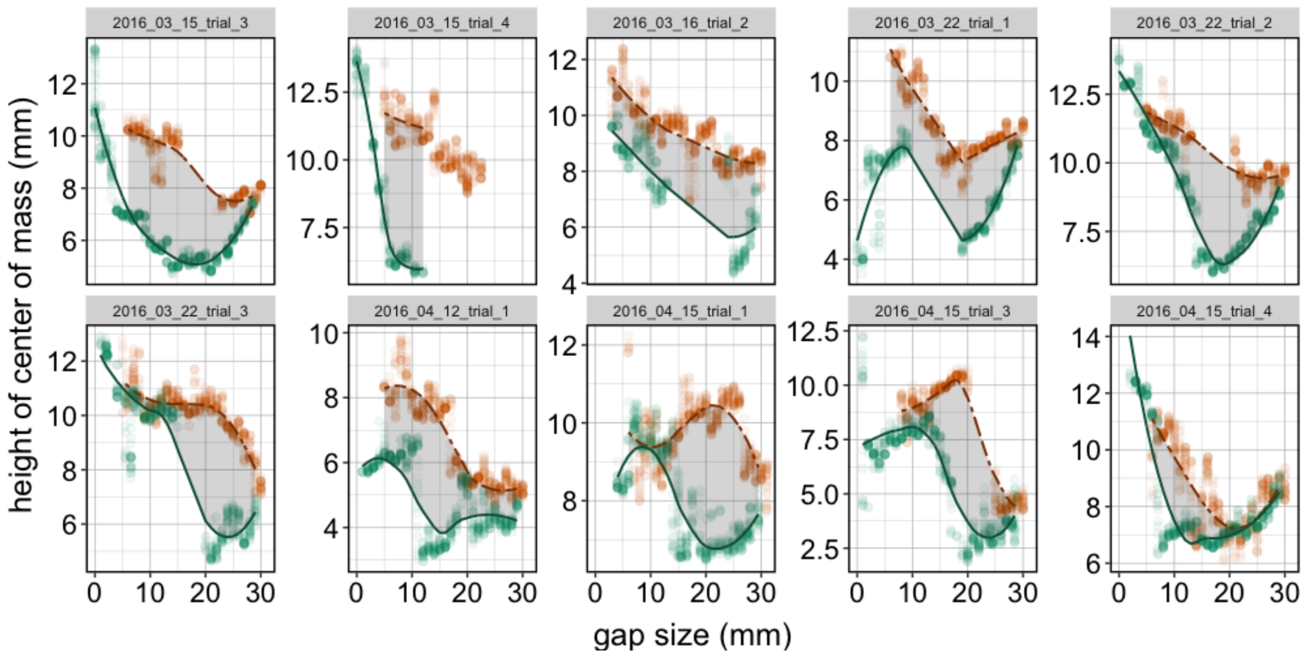

Supplementary Figure 8. Hysteresis in the height of the center of mass (droop) for all trials. Trials consistently show hysteresis, with a bridge at a particular gap size hanging lower during the contraction than the expansion phase. Points show individual measurements, taken every second; periods when the bridge was broken or recovering are excluded. Lines are smoothed LOESS (local regression) for the expansion (orange points, dashed orange line) and contraction (green points, solid green line) phases. The area between the smoothed lines (shaded gray) shows the extent of hysteresis. Points are jittered to improve clarity.

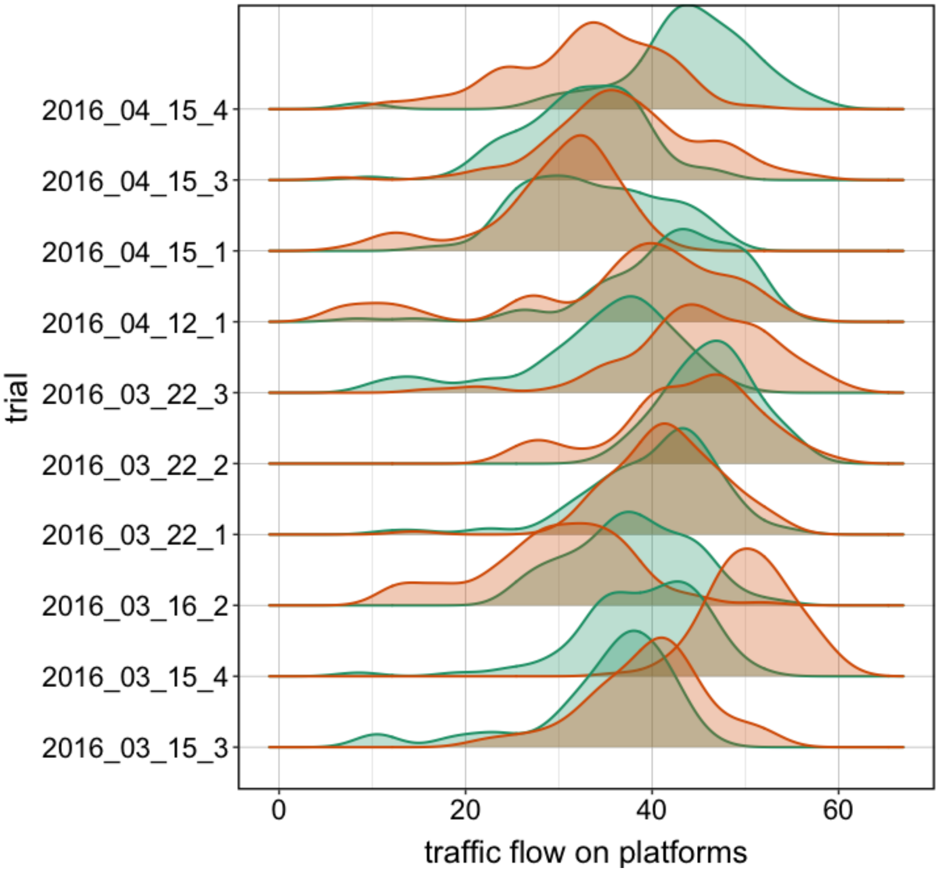

Supplementary Figure 9. Traffic does not co-vary with experimental phase. The distributions of total traffic on platforms during the expansion (orange) and contraction (green) overlap substantially for all trials. Our traffic measure is based on optic flow as described in Appendix A.5, which measures the amount of movement from one video frame to the next, in units of pixels per frame. Traffic varied during trials, but not predictably with respect to experimental phase. Some trials had heavier traffic during the expansion, some had heavier traffic during the contraction, while others were more consistent.

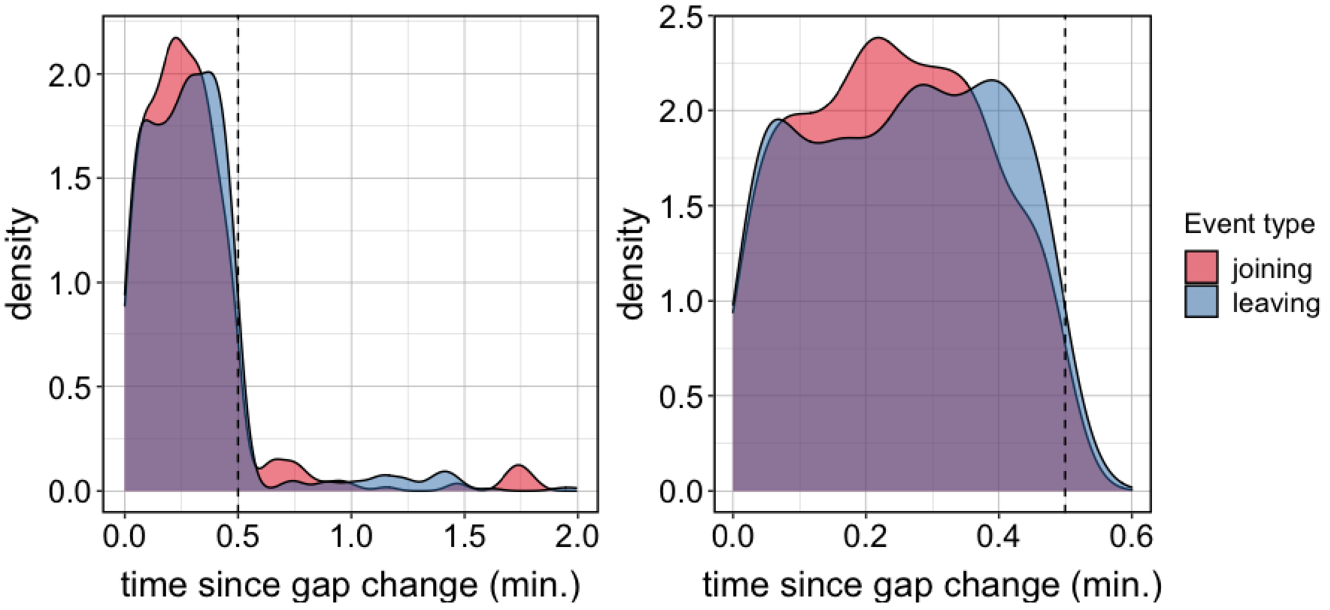

Supplementary Figure 10. Events are not directly prompted by gap changes. The distribution of joining and leaving events is even throughout gap intervals. A) Some intervals last longer than 30 seconds if the bridge was non-functional. B) Excluding events occurring after the 30 second mark for longer intervals, the distributions for both joining and leaving are relatively uniform. Events are not more likely to occur immediately after gap size changes. The number of events appears to drop shortly before 30 seconds; this is an artifact of the distribution smoothing.

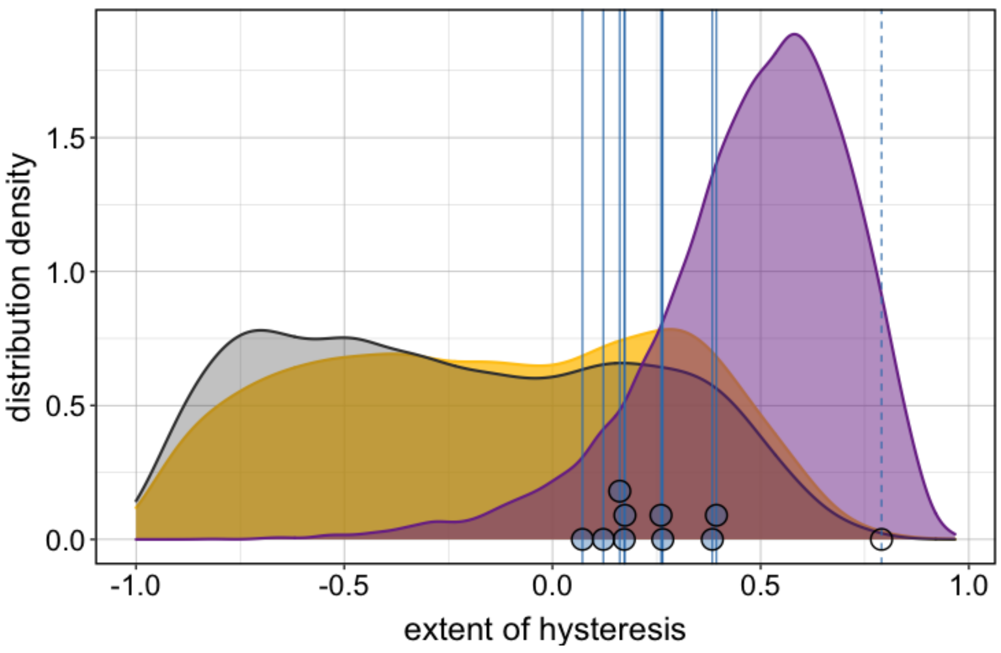

Supplementary Figure 11. Extents of hysteresis for all models (distributions) and data (points). Distributions of hysteresis extents over 10,000 simulations for the baseline (gray), delay (yellow), and accumulator (purple) models. The hysteresis extents in experiments are shown as blue points with corresponding vertical lines. Dashed line and open point indicate the trial in which the bridge was broken or recovering the majority of the time. Extent of hysteresis is a unitless proportion showing the degree to which the number of ants in bridges differs between the experimental phases.

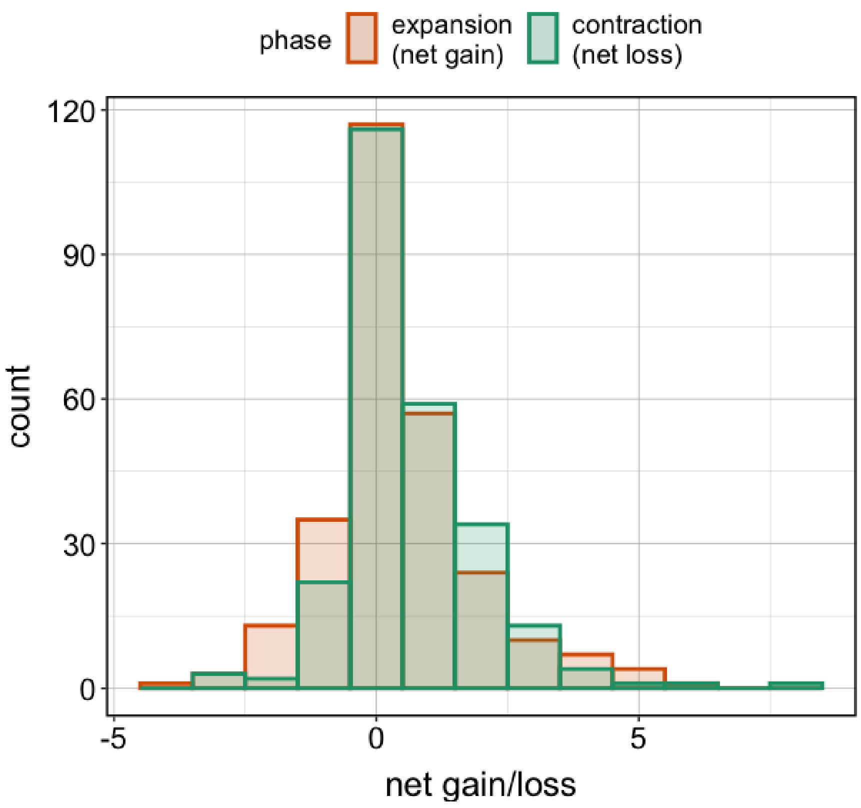

Supplementary Figure 12. Asymmetry in net gain/loss of ants from bridges. The distribution of the net *gain* of ants (number joining - number leaving) during each 30 sec gap interval in the expansion (orange) and the distribution of the net *loss* of ants (number leaving - number joining) in the contraction (green). The distributions are very slightly asymmetrical; experimental bridges tended to lose ants somewhat more rapidly in the contraction than they gained them in the expansion. This slight asymmetry accounts for the fact that the data-driven linear models produce, on average, negative hysteresis in simulated bridges (main text Figures 4A and B), as well as the fact that simulated bridges undergoing vibration-like changes tend to lose ants on average (main text Figures 4D and E).

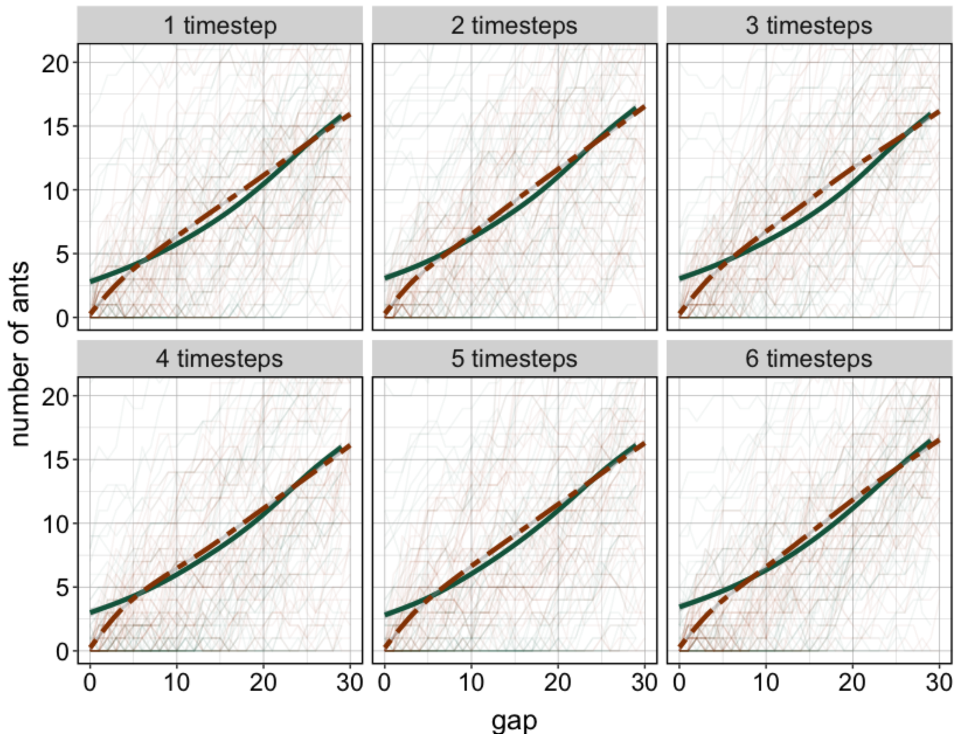

Supplementary Figure 13. Simulation results for linear delay model for all delay durations. Shows the same simulation data as Figure 4B in the main text, but for delay durations of 1 to 6 timesteps. Results of 1,000 simulations are shown in each panel.

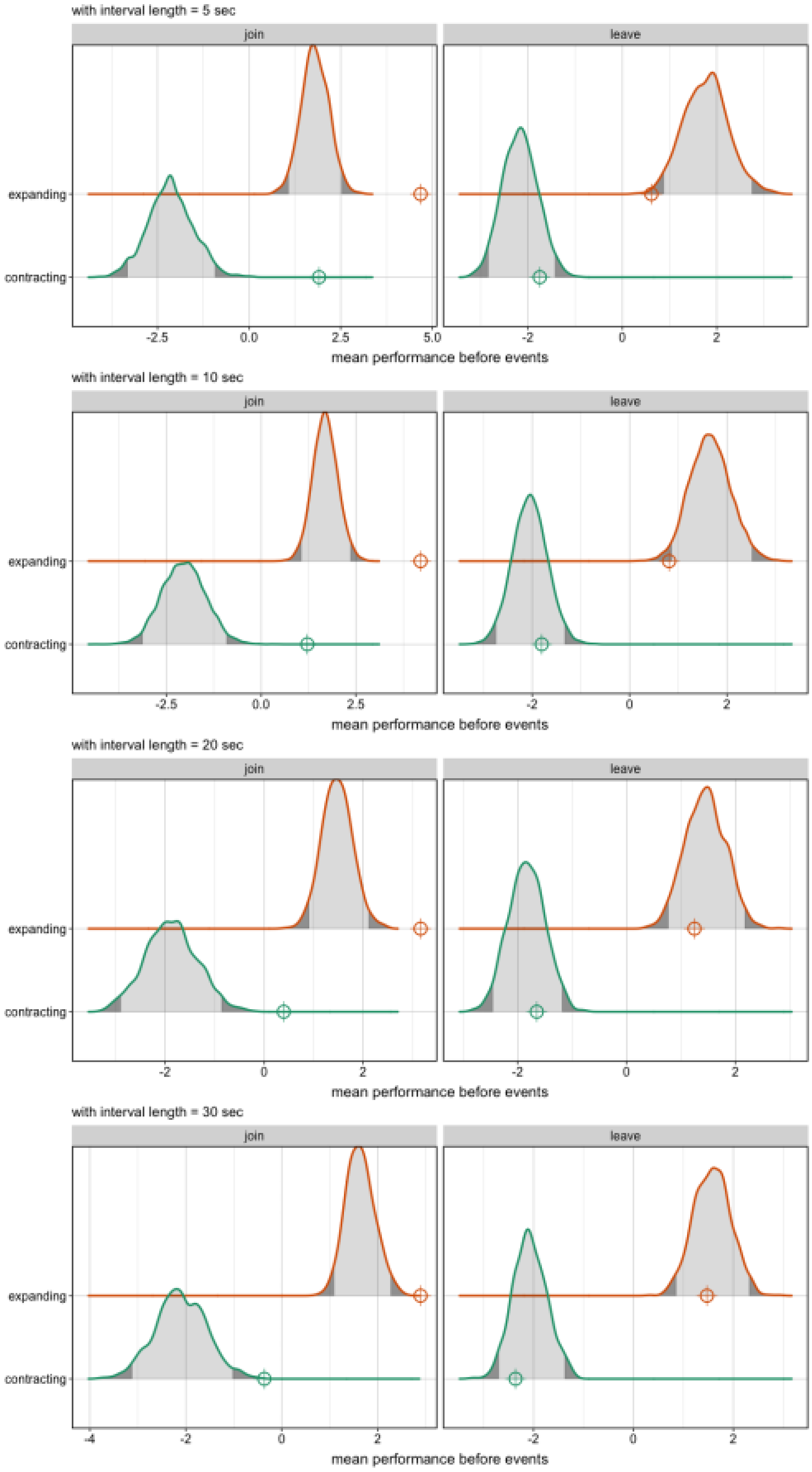

Supplementary Figure 14. Sensitivity analysis for effects of performance on events. Shows the same analysis as in Figure 7 in the main text, but using additional intervals over which performance is measured (from top to bottom: 5 seconds, 10 seconds (main text version), 20 seconds, and 30 seconds). The conclusion that high performance prompts joining is not sensitive to the interval length, though the effect is weaker as the interval increases.

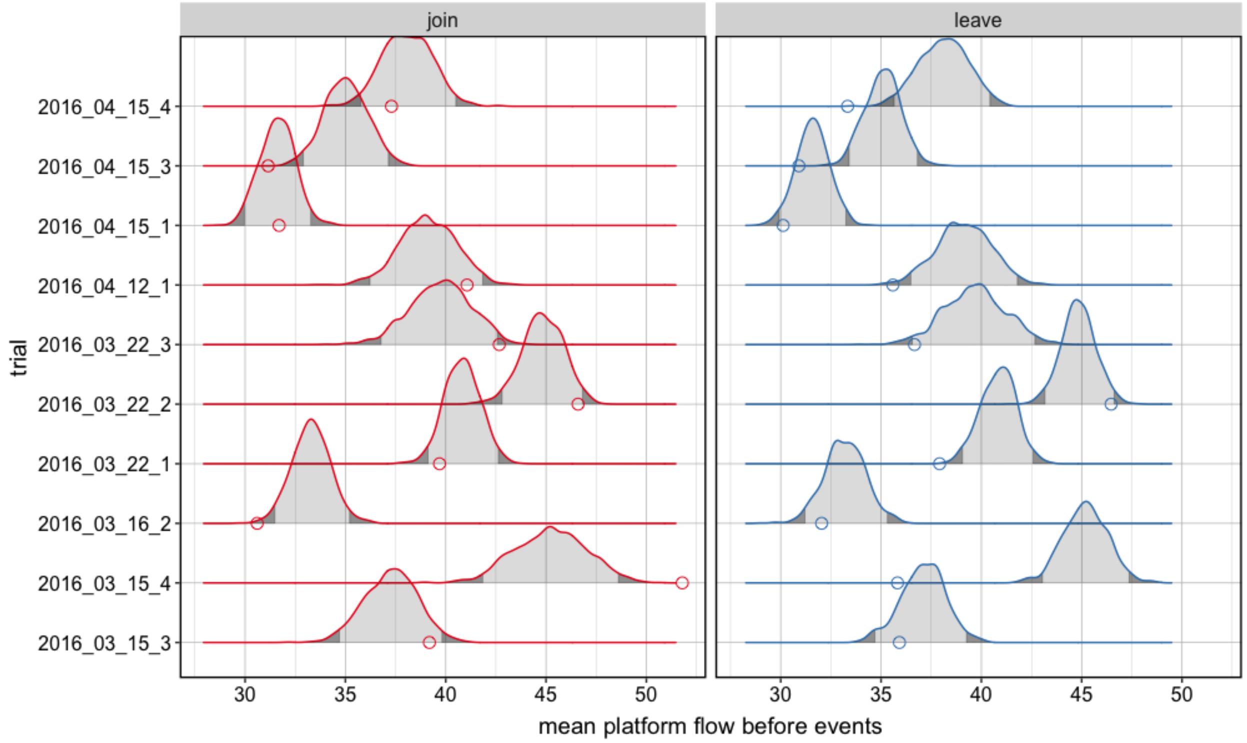

Supplementary Figure 15. Overall traffic before events. Mean traffic (optic flow) over platforms immediately preceding events in each trial (open circles) and mean traffic at randomized times from 10,000 simulations (distributions). The y-axis shows trial ID. While we expect overall traffic to affect joining and/or leaving events, using optic flow on platforms as a measure, such patterns are not consistent across trials, and are less clear than the signal of high performance causing joining.

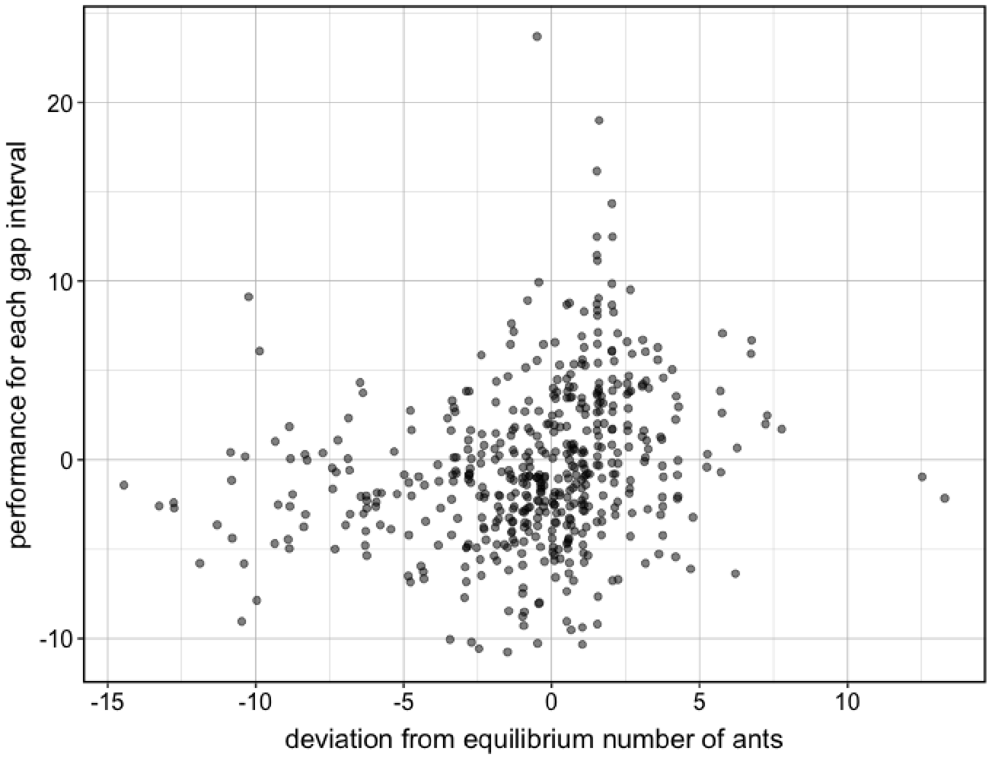

Supplementary Figure 16. High deficit is not associated with low performance. Each point corresponds to a 30 second gap interval. Performance measured using optic flow, as described in main text and Appendix A. The deviation from equilibrium number of ants is measured using the equilibrium packing density of 0.51 ants per mm of gap length.

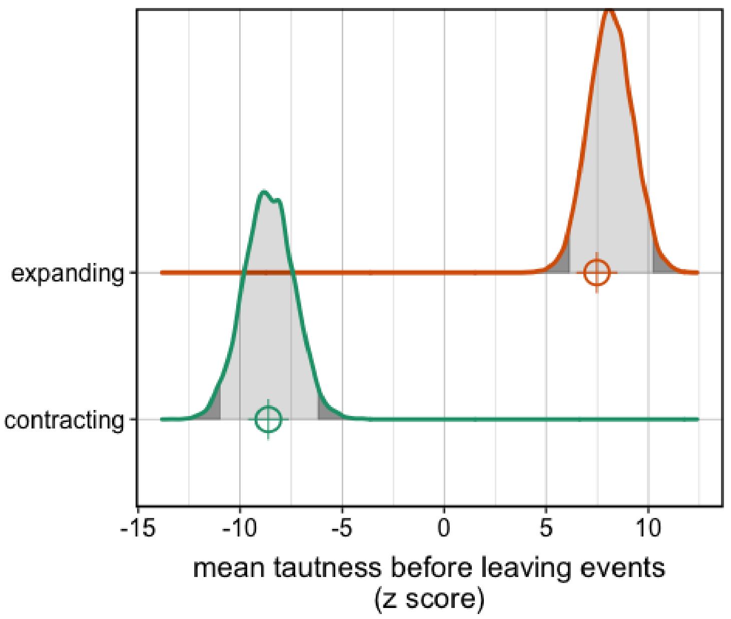

Supplementary Figure 17. For entire structures, tautness before leaving events is not different than expected by chance. The effect of tautness (height of the center of mass, as a z score for each trial) on leaving events was analyzed with a resampling approach. Distributions show the mean performance during the 10 seconds preceding events at random times, from 10,000 simulations leaving events during the expansion (orange) and contraction (green). Dark gray shading indicates the most extreme 5% of each distribution (below 2.5% and above 97.5%). The corresponding means from the experiments are shown as points (open circles with cross-hairs). Tautness of bridges preceding leaving events is not different than expected by chance in either phase, though as discussed in main text, variation of mechanical factors within a bridge is likely important (location of an ant in the structure, network of grasping points).

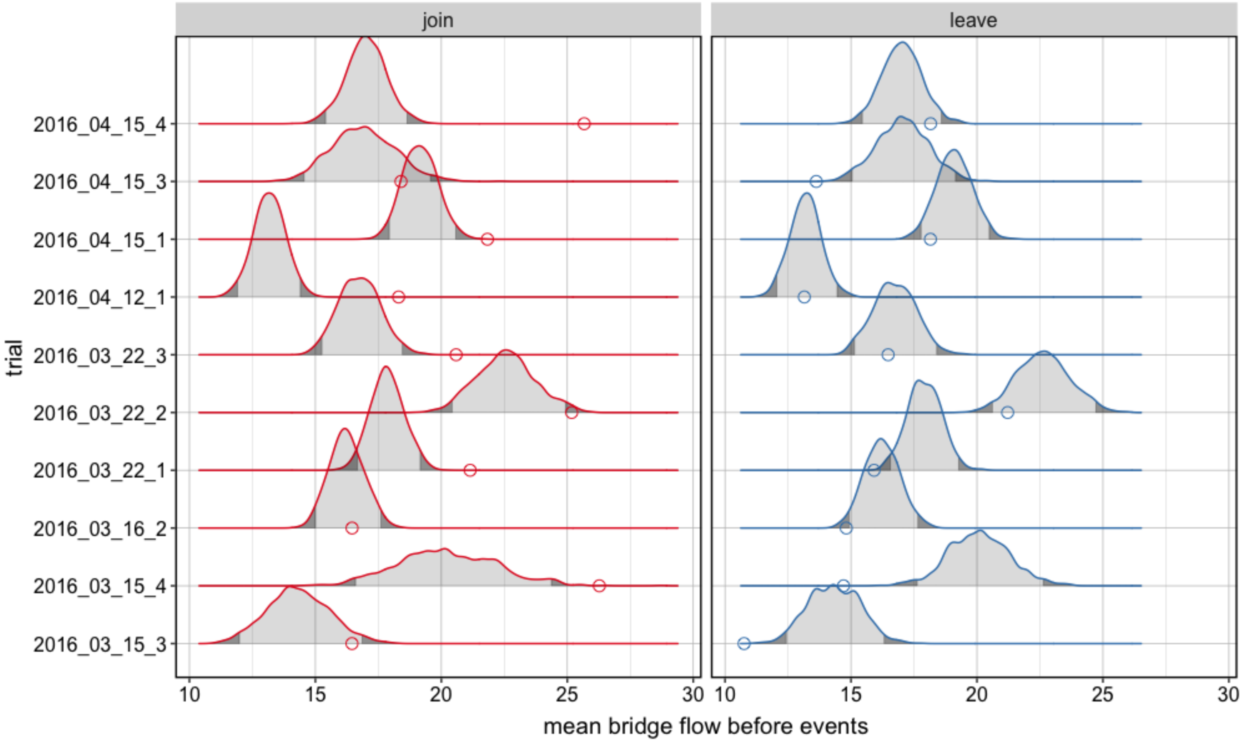

Supplementary Figure 18. Traffic on bridge before events. Mean traffic (optic flow) over bridges immediately preceding events in each trial (open circles) and mean traffic at randomized times from 10,000 simulations (distributions). The y-axis shows trial ID. Joining events are preceded by high bridge traffic. This makes intuitive sense, as ants cannot join a bridge unless they are currently walking on it. Furthermore, leaving events are typically preceded by low traffic. This fits with the expected pattern that ants will leave a bridge if they are not being walked on.

Supplementary References

1. Gombin, J., Vaidyanathan, R. & Agafonkin, V. *concaveman: A Very Fast 2D Concave Hull Algorithm*. (2020).
2. Hamilton, N. *smoother: Functions Relating to the Smoothing of Numerical Data*. (2015).
3. Pebesma, E. Simple Features for R: Standardized Support for Spatial Vector Data. *The R Journal* **10**, 439–446 (2018).
4. Wickham, H. *et al.* Welcome to the tidyverse. *K* **4**, 1686 (2019).
5. Farnebäck, G. Two-Frame Motion Estimation Based on Polynomial Expansion. in *Image Analysis* (eds. Bigun, J. & Gustavsson, T.) 363–370 (Springer, 2003). doi:10.1007/3-540-45103-X_50.
6. Bradski, G. The OpenCV Library. *Dr. Dobb's Journal of Software Tools* (2000).
