# Supplementary material for: Hysteresis stabilizes dynamic control of self-assembled army ant constructions
Source: Nat Commun. 2022 Mar 4;13:1160. doi: 10.1038/s41467-022-28773-z (PMC8897433; doi:10.1038/s41467-022-28773-z)
Supplement: Supplementary file 4 — Reporting Summary [file 41467_2022_28773_MOESM4_ESM.pdf]

nature portfolio

Corresponding author(s): Helen McCreery

Last updated by author(s): Jan 29, 2022

Reporting Summary

Nature Portfolio wishes to improve the reproducibility of the work that we publish. This form provides structure for consistency and transparency in reporting. For further information on Nature Portfolio policies, see our Editorial Policies and the Editorial Policy Checklist.

Statistics

For all statistical analyses, confirm that the following items are present in the figure legend, table legend, main text, or Methods section.

n/a	Confirmed	
☐	☒	The exact sample size (n) for each experimental group/condition, given as a discrete number and unit of measurement
☐	☒	A statement on whether measurements were taken from distinct samples or whether the same sample was measured repeatedly
☐	☒	The statistical test(s) used AND whether they are one- or two-sided<br>*Only common tests should be described solely by name; describe more complex techniques in the Methods section.*
☐	☒	A description of all covariates tested
☐	☒	A description of any assumptions or corrections, such as tests of normality and adjustment for multiple comparisons
☐	☒	A full description of the statistical parameters including central tendency (e.g. means) or other basic estimates (e.g. regression coefficient) AND variation (e.g. standard deviation) or associated estimates of uncertainty (e.g. confidence intervals)
☐	☒	For null hypothesis testing, the test statistic (e.g. F, t, r) with confidence intervals, effect sizes, degrees of freedom and P value noted<br>*Give P values as exact values whenever suitable.*
☒	☐	For Bayesian analysis, information on the choice of priors and Markov chain Monte Carlo settings
☒	☐	For hierarchical and complex designs, identification of the appropriate level for tests and full reporting of outcomes
☐	☒	Estimates of effect sizes (e.g. Cohen's d, Pearson's r), indicating how they were calculated

Our web collection on statistics for biologists contains articles on many of the points above.

Software and code

Policy information about availability of computer code

Data collection	No software was used to collect the video data used for this study.
Data analysis	As described in the manuscript, the following types of commercially available software were used for this study: Matlab (version R2017b), R (version 3.6.3), Open CV (version 4.3), and R packages "concaveman" version 1.1.0, "smoother" version 1.1, "sf" released 2018, "tidyverse" released 2019, "ggridges" version 0.5.2, "gridExtra" version 2.3, "fitdistrplus" released 2015, "MASS" released 2002, and "DHARMa" version 0.3.2.0. We also developed custom code for analysis using this software, to extract data from videos of experiments and to analyze the data. All of the code we used for the study is available on figshare at https://figshare.com/s/bf97642c8887c2c1af71. The code is published and cited in the manuscript with DOI: 10.6084/m9.figshare.13337255.v1.

For manuscripts utilizing custom algorithms or software that are central to the research but not yet described in published literature, software must be made available to editors and reviewers. We strongly encourage code deposition in a community repository (e.g. GitHub). See the Nature Portfolio guidelines for submitting code & software for further information.

Data

Policy information about availability of data

All manuscripts must include a data availability statement. This statement should provide the following information, where applicable:

- Accession codes, unique identifiers, or web links for publicly available datasets
- A description of any restrictions on data availability
- For clinical datasets or third party data, please ensure that the statement adheres to our policy

Source data are provided with this paper. Data are available on figshare at https://figshare.com/s/bf97642c8887c2c1af71. This is a private, non-searchable link. We will publish the linked files upon publication. Full raw videos of experiments are available upon request.

Field-specific reporting

Please select the one below that is the best fit for your research. If you are not sure, read the appropriate sections before making your selection.

☐ Life sciences ☐ Behavioural & social sciences ☒ Ecological, evolutionary & environmental sciences

For a reference copy of the document with all sections, see nature.com/documents/nr-reporting-summary-flat.pdf

Ecological, evolutionary & environmental sciences study design

All studies must disclose on these points even when the disclosure is negative.

Study description	We conducted field experiments on Eciton hamatum army ant foraging trails, causing the ants to construct self-assembled bridges and repeatedly forcing adjustments in the bridges to understand how they respond. There were no separate treatments for this study. All trials were the same, and we video recorded trials to extract behavioral data. We used mechanistic and statistical models to understand how the dynamics we observed emerged. Each experimental unit is a trial, with no subdivision within, and we had 10 complete replicate trials. However, each trial contained a great deal of data (thousands of data points), as we collected data through time. To avoid any pseudoreplication, we either conducted analyses at the trial level, or we specifically accounted for possible trial effects.
Research sample	Each research sample for our experimental data was a foraging trail of wild Eciton hamatum army ants on Barro Colorado Island in Panama. We set up experiments on robust foraging trails, with heavy ant traffic flow. While it was not possible to definitively exclude ants represented in one trial from subsequent trials, each foraging trail in total contained many thousands of ants, which together with the rapid traffic flow (ants in one trial continued traveling rapidly along the trail either during or immediately after the trial ended) ensured that individuals were not repeated across trials. We chose our study site (Barro Colorado Island) to maximize our chances of encountering our study species, as the island is known to have a high density of Eciton hamatum colonies.<br><br>In our experiments, we sampled Eciton hamatum foragers and other workers (across all castes: minors, medias, submajors, and majors) that were traveling on foraging trails. The rationale for this is that we wanted a sample that was representative of the population of Eciton hamatum ants traveling on the foraging trails. All workers traveling on the experimental segment of the foraging trail during our experiments participated in the study, we did not exclude or select particular individuals. All ant workers are female, therefore all animals in our study were female. Individual Eciton workers are thought to live for approximately 1 year, so we estimate that all our study animals were less than 1 year old. We did not perform any manipulations on study animals - our only manipulations were to move the foraging trail onto our apparatus (by guiding the ants to reroute using material with their pheromone) and changing the size of the gap in the apparatus.
Sampling strategy	We did not decide sample size statistically, but got as many complete trials as we could in over 2 months in the field. In order for a trial to succeed, we needed steady, heavy traffic flow on the ants' foraging trails. Thus in many cases trials were started but could not be completed before traffic dropped. This limited our sample size, but we were able to ensure in our data analyses that our sample size was more than sufficient for all of the conclusions we draw from the data.
Data collection	Helen McCreery collected the experimental data in the field. As army ant colonies move every night, each morning data collection began by searching for a robust army ant foraging trail. After finding a trail, Helen set up the experimental apparatus (consisting of 3D printed platforms as described in the manuscript). Helen moved the foraging trail onto the apparatus, so that a steady stream of ants traveled from the natural trail onto one platform, across to the other platform, and back onto the natural trail. After setting up multiple cameras to record experiments, Helen then opened a gap between the platforms and gradually increased the gap size so that crossing ants self-assembled into a bridge. Trials progressed with the bridge expanding to 3 cm and then back to no gap.
Timing and spatial scale	All data were collected on Barro Colorado Island in Panama (covering approximately 15 square kilometers). We chose this location to maximize our changes of encountering the study species, as Barro Colorado Island is known to have a high density of Eciton hamatum colonies. Data were collected in March and April 2016. Eciton hamatum colonies actively forage year-round, but become less active during heaving rain events. We chose March and April because this is the dry season on Barro Colorado Island. Not only does this mean the ants are more likely to be actively foraging on any given day, but it also made it more likely that we could actively study them, as rain made it difficult to collect high quality videos of experiments. Attempts were made to conduct experiments at least 6 days per week, though as noted above, experiments required at least 30 minutes of strong, consistent traffic on ant foraging trails which were out of our control, so on many days complete experiments were not possible.<br><br>We attempted to collect data continuously throughout March and April in 2016. However, trials could only be completed when the natural traffic over the ants' foraging trails was not only relatively high, but was also relatively consistent through time (for at least an hour). We were able to collect one or more complete trials on March 15, March 16, March 22, April 12, and April 15, 2016. We decided to stop collecting after mid April because we had collected a reasonable, if relatively small, set of complete trials, and more saliently because we had run out of time in our planned field work.
Data exclusions	One trial was excluded from some analyses of individual behavior (joining and leaving events. We excluded it because in trial, the self-assembled ant bridge was either broken or recovering from a break for the majority of the trial. We could not be confident in the joining and leaving rates during these periods. We also excluded periods of time within trials during which the bridge was broken from all analyses.
Reproducibility	Collective dynamics were remarkably similar across the 10 complete trials. Incomplete trials were those during which ant traffic dropped and thus the ants abandoned the bridge, we never excluded trials based on results different from expectations or from other trials. All attempts to repeat the experiment were successful when ant traffic was sufficient, as discussed in the manuscript.

Randomization	This is not relevant to our study, because we had no separate treatments. All trials were conducted following the same procedure and with the same treatment.
Blinding	Blinding was not relevant to our study, as there were no different treatments.

Did the study involve field work? ☒ Yes ☐ No

Field work, collection and transport

Field conditions	Conditions were typical of a tropical rainforest during the dry season. Temperature was between 85 and 95 degrees F each day, and humidity was above 90%. We did not conduct experiments when rainfall was very heavy, as army ants do not typically forage in these conditions.
Location	Barro Colorado Island, Panama. Longitude 9.15, latitude -79.85, elevation 120 m. All research was conducted on dry land, in tropical rainforest leaflitter.
Access & import/export	All research was conducted within the bounds of the Smithsonian Tropical Research Institute (STRI) research station on Barro Colorado Island. We applied for and received all necessary approvals from STRI. Specifically, we received our project approval on February 16, 2016, for project number 3920. Beyond this approval, our project did not require any permits. As our data consisted of videos of experiments, we did not transport any samples at all.
Disturbance	The disturbance caused by these experiments was minimal, as we did not remove the army ants from their natural habitat, conducing all experiments in situ. We temporarily disturbed foraging trails in order to set up our experiments. However, our experiments relied on the foraging trails being functional, so any disturbance was necessarily short-lived.

Reporting for specific materials, systems and methods

We require information from authors about some types of materials, experimental systems and methods used in many studies. Here, indicate whether each material, system or method listed is relevant to your study. If you are not sure if a list item applies to your research, read the appropriate section before selecting a response.

Materials & experimental systems

n/a	Involved in the study	
☒	☐	Antibodies
☒	☐	Eukaryotic cell lines
☒	☐	Palaeontology and archaeology
☐	☒	Animals and other organisms
☒	☐	Human research participants
☒	☐	Clinical data
☒	☐	Dual use research of concern

Methods

n/a	Involved in the study	
☒	☐	ChIP-seq
☒	☐	Flow cytometry
☒	☐	MRI-based neuroimaging

Animals and other organisms

Policy information about studies involving animals; ARRIVE guidelines recommended for reporting animal research

Laboratory animals	This study did not involve laboratory animals.
Wild animals	We performed this study on wildtype Eciton hamatum ants (all female workers) in their natural habitat, within existing foraging trails. The lifespan of Eciton workers is thought to be 1 year; all study animals were therefore up to 1 year of age. No animals were captured, transported or held in captivity.
Field-collected samples	As our data were collected by video recording experiments, our study did not involve samples collected from the field.
Ethics oversight	The Smithsonian Tropical Research Institute provided guidance and approval on the study protocol.

Note that full information on the approval of the study protocol must also be provided in the manuscript.
